# The impact of cardiopulmonary resuscitation (CPR) manikin chest stiffness on motivation and CPR performance measures in children undergoing CPR training—A prospective, randomized, single-blind, controlled trial

**DOI:** 10.1371/journal.pone.0202430

**Published:** 2018-08-16

**Authors:** David Weidenauer, Thomas Hamp, Christoph Schriefl, Caroline Holaubek, Markus Gattinger, Mario Krammel, Markus Winnisch, Ana Weidenauer, Gerald Mundigler, Irene Lang, Wolfgang Schreiber, Fritz Sterz, Harald Herkner, Hans Domanovits

**Affiliations:** 1 Department of Internal Medicine II, Medical University of Vienna, Vienna, Austria; 2 Department of General Anesthesia and Intensive Care, Prehospital Emergency Medicine Research Group, Medical University of Vienna, Vienna, Austria; 3 Department of Emergency Medicine, Medical University of Vienna, Vienna, Austria; 4 Department of Cardiothoracic and Vascular Anesthesia, Medical University of Vienna, Vienna, Austria; 5 Medical University of Vienna, Vienna, Austria; 6 Municipal Ambulance Service of Vienna, Vienna, Austria; 7 Department of Trauma Surgery, Medical University of Vienna, Vienna, Austria; 8 Department of Psychiatry and Psychotherapy, Medical University of Vienna, Vienna, Austria; Waseda University, JAPAN

## Abstract

**Background:**

Cardio-pulmonary-resuscitation (CPR) training starting at the age of 12 years is recommended internationally. Training younger children is not recommended because young children lack the physical ability to perform adequate CPR and discouragement to perform CPR later is apprehended. The aim of this study was to answer the following questions: Are younger children discouraged after CPR training? Is discouragement caused by their lack in physical ability to perform adequate chest compressions on a standard manikin and would the use of manikins with a reduced resistance affect their motivation or performance?

**Methods:**

We investigated the motivation and CPR performance of children aged 8–13 years after CPR training on manikins of different chest stiffness in a prospective, randomized, single-blind, controlled trial. 322 children underwent randomization and received 30 minutes CPR training in small groups at school. We used two optically identical resuscitation manikins with different compression resistances of 45kg and 30kg. Motivation was assessed with a self-administered questionnaire. Performance was measured with the Resusci^®^Anne SkillReporter^™^.

**Findings:**

Motivation after the training was generally high and there was no difference between the two groups in any of the questionnaire items on motivation: Children had fun (98 vs. 99%; P = 0.32), were interested in the training (99 vs. 98%; P = 0.65), and were glad to train resuscitation again in the future (89 vs. 91%; P = 0.89). CPR performance was generally poor (median compression score (8, IQR 1–45 and 29, IQR 11–54; P<0.001) and the mean compression depth was lower in the 45kg-resistance than in the 30kg-resistance group (33±10mm vs. 41±9; P<0.001).

**Conclusions:**

Compression resistances of manikins, though influencing CPR performance, did not discourage 8 to 13 year old children after CPR training. The findings refute the view that young children are discouraged when receiving CPR training even though they are physically not able to perform adequate CPR.

## Introduction

Sudden out-of-hospital cardiac arrest (OHCA) with unsuccessful cardiopulmonary resuscitation (CPR) is the third leading cause of death in industrialized nations [1]. The bystander has a key function in the chain of survival, as the chance to survive a cardiac arrest is up to 2 to 4 times more likely with bystander CPR [2]. The rates of bystander CPR are vary widely between different areas, communities and countries ranging from 0 to 100% but are often < 20% in European and North American countries [3,4], and after OHCA, the overall survival remains poor with rates from 2 to 10% in most industrialized countries [5–7]. “Increasing the percentage of the population trained in CPR is an integral part of an overall strategy to improve response to OHCA” according to a statement by the American Heart Association [8].

A mandatory CPR training at school has the highest impact for improving the bystander CPR rate [2,9–11]. To have a less inhibited approach to resuscitation training, and to be more self-confident in case of an emergency, the training should start before puberty and should be repeated at regular intervals during the school career [12,13]. However, the ability to achieve an adequate depth of chest compression depends on age and body weight [14]. To overcome the standard resistance and compress the chest 50mm deep during training with an adult resuscitation manikin, a pressure of a weight of 45kg is necessary. Studies have shown that 13 years, where most children reach a weight of 50kg, is the minimum age to be able to achieve a CPR quality similar to the one of adults and younger children are usually not able to perform adequate chest compressions [14,15].

Hence the International Liaison Committee on Resuscitation (ILCOR) recommends two hours of CPR training annually from the age of 12 years in all schools worldwide [16]. The WHO has endorsed this position statement [17].

Nevertheless, there is evidence that children younger than 8 years—although not being physically able to perform adequate chest compressions—are already able to assess consciousness and breathing, to deploy the automatic extern defibrillator (AED) and to give sufficient information during an emergency telephone conversation; furthermore, they are easily motivated and learn quickly [18–21]. Those who are not already physically developed enough to compress the chest adequately can at least learn the principles of chest compression [13,14]. Moreover, they can teach their relatives and friends Basic Life Support (BLS), which has been shown to induce a more positive attitude towards bystander CPR [22]. Despite these advantages, the ILCOR, the American Heart Association (AHA) and other members of ILCOR argue against CPR training in school children younger than 12 years, as of yet. The AHA emphasizes there might be a risk of discouragement or lack of interest in younger children when receiving CPR training on adult manikins, because children are not able to compress the chest deep enough during training and as a consequence might be discouraged, demotivated and disinterested [8].

However, to our knowledge, no studies have specifically addressed this important question—whether or not children are discouraged, demotivated, and disinterested after receiving CPR training on standard adult manikins on which young children inevitable would perform poorly. Many children are excluded from resuscitation training due to this lack of evidence. Therefore, we conducted a study to investigate the motivation of young school children after receiving CPR training and also assessed their CPR performance after the training. We conducted the training on two optically identical manikins with different compression resistances to specifically assess the impact of manikin stiffness on children`s motivation and CPR performance after the resuscitation training.

## Methods

We conducted this study as a prospective, randomized, single-blind controlled trial in accordance with the Declaration of Helsinki (World Medical Association) and the Good Clinical Practice Guidelines (International Conference on Harmonization).

### Study population

This study was approved by the Ethics Committee of the Medical University of Vienna (Reg. Nr.: 1490/2014) and we included 8 to 11 year old children and also 12 to 13 year old children to allow a comparison between motivation in children of a guideline conform age and younger children in a subgroup analysis. We included children from three schools in Vienna, Austria and enrolled them after obtaining written consent from all parents.

Children were excluded if written consent could not be obtained and if they did not attend the entire training.

### Study design and randomisation

After enrolment, we recorded children’s date of birth, sex, weight, height, and whether or not they have ever practiced cardiorespiratory resuscitation before. We randomized the children into two groups, the soft manikin group (intervention group) and standard manikin group (control group). Randomization was performed as complete randomization with a computer-generated randomization list derived from an online randomisation software (www.randomizer.at, Institute for Medical Informatics, Statistics and Documentation, Medical University of Graz, Graz, Austria).

#### Study flow

The children in each group where then further divided into smaller groups of 5 to 6 children of the same age to allow an optimal learning environment and closer supervision by the trainer. The children then received 30 minutes of resuscitation training on a Resusci Anne QCPR training manikin in which the following skills were taught theoretically and practically: recognition of arrest, phoning for help, chest compressions only CPR (depth, rate, and degree of recoil). Every child was obliged to perform at least two short runs of chest compressions during the training and children received continuous feedback on hand positioning, compression depth, compressions rate, and the degree of recoil.

The training was carried out by certified first aid trainers that were randomly allocated to the groups. These trainers regularly perform first aid trainings in schools in Austria and therefore have significant experience in training children. The trainers were blinded to the resistance of the manikin and to the group assignment of the children they trained.

In the soft manikin group, a training manikin with reduced resistance (30kg) was used, while in the standard manikin group an optically identical standard resistance manikin (45kg) was used.

Children were not aware of the existence of the difference in resistance of the manikins and were told that the splitting into groups was done in order to prevent distraction from the actual training.

After the children had completed the training, we asked them to perform two minutes of continuous chest compressions on the same manikin they had been trained on and recorded their performance with the Resusci^®^Anne Wireless SkillReporter^™^ (Laerdal). This system has already been used in several studies investigating CPR quality [23,24] and is able to detect and record chest compressions characteristics in real time (rate, depth, leaning and hands position) and also calculates the so called “compressions score” which represents the overall chest compression quality and ranges from 0–100. The following parameters are used to calculate the compression score: compression depth, compression rate, incomplete release, number of compressions per cycle, number of compressions per cycle and hand position.

We used the PC Software Session Viewer by Laerdal (Version 5.1), which is a stand-alone application for recording, viewing and editing exported SimView debriefing files, SimPad log files and BLS result files. The system was configured according to the AHA and ERC 2015 guidelines: correct depth 50–60 mm and correct compressions rate 100–120 per min [25,26].

More detailed information on software scoring could be retrieved in the manufacturer’s website (http://cdn.laerdal.com/downloads/f3943/Att_2_to_00021778.pdf).

We conducted the evaluations in the same room, with the same group of children, with the same manikin in order to have the same situation as during the training. No feedback was permitted during the test by the trainer or by the feedback system and the children did not know that their performance was being recorded.

After testing, each child filled out a questionnaire with four possible answers per question in a separate room. The questionnaire was developed by the members of the study team and consisted of six questions on joy during the training, interest in the training, willingness to repeat the training in the future, difficulty of performing chest compressions, and self-rating (S1 Questionnaire). There were four possible answers to each question (i.e. it was a lot of fun—it was fun—I had little fun—I did not like it at all) to avoid the error of central tendency frequently seen with three- or five-point scales.

**Fig 1:**
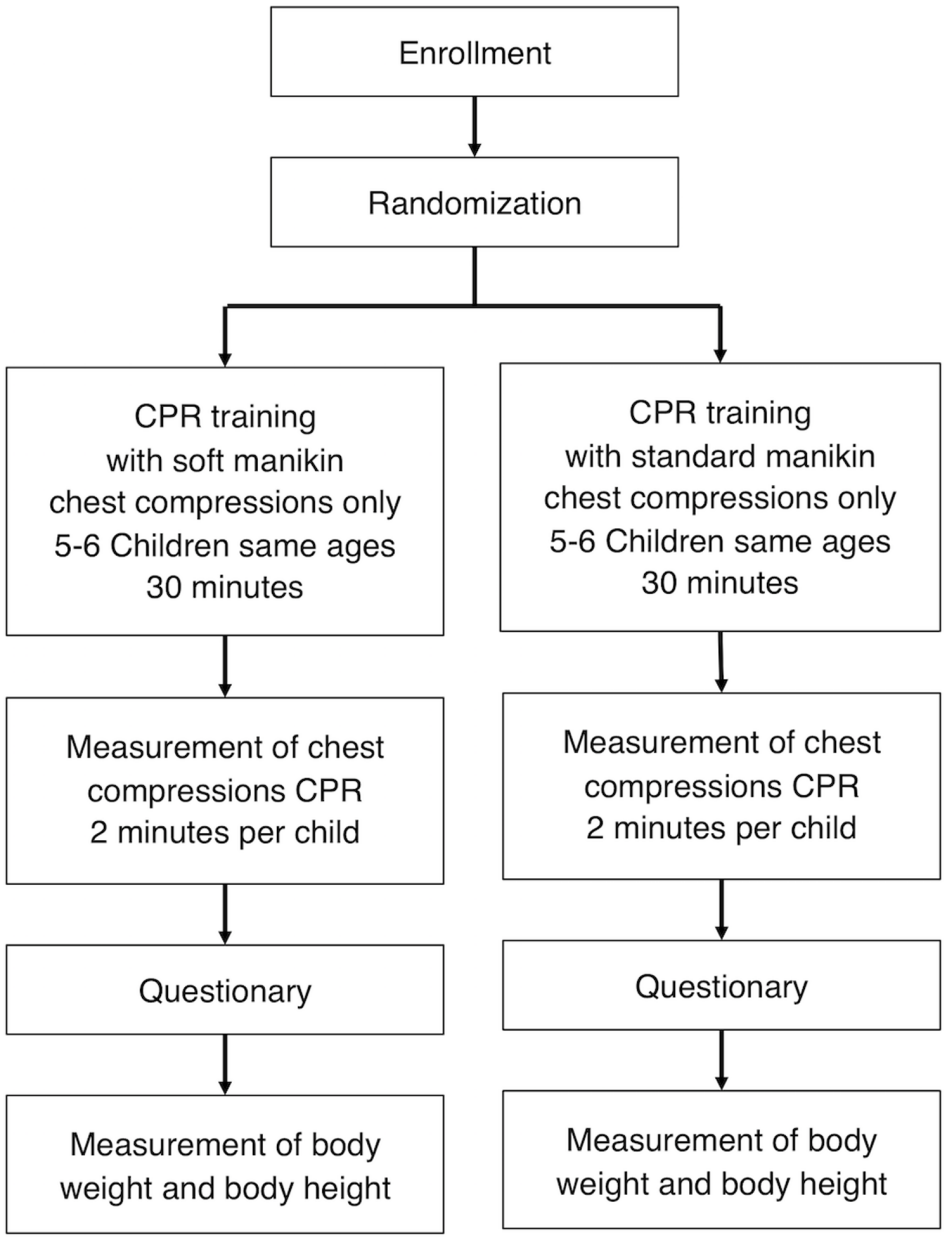
Study interventions.

In addition, body weight and height of each child were measured and completed study participation. Fig 1 illustrates the screening and randomization process and the study interventions.

### Statistical methods

We present categorical data as count and relative frequency. Continuous data are presented as median with 25 to 75% quartiles. We expected that we would formally need 228 children to detect a difference of 15% in the dichotomized answer categories (best versus other) at a power of 80% and a significance level of 5% for each of the cardinal questions. Adding 10% for item non-response and a design factor of 1.2 for potential clustering this resulted in a total sample size of 300 children.

We tabulated baseline and demographic variables to assess randomization success. We did not use formal hypothesis testing for this aspect. For the analysis of the object “motivation”, we compared the answer categories to experimental group allocation using contingency tables and the Fisher’s exact test for hypothesis testing (H_0_ = no difference). To assess the influence of covariate we dichotomized answer categories as the best answer versus all other answers. We used multivariable logistic regression models to assess the influence of covariate on the main effects. We used these models to test for first order interactions. Given a potential non-independence of data because the children were nested within classrooms or nested within schools we calculated the effects using random coefficient effects models with cluster variables and calculated standard errors using sandwich estimators. We used the Wald test for hypothesis testing within the models. For the analysis of CPR performance, we tabulated data according to experimental group allocation, present boxplots and used the Mann Whitney U-test to test the H_0_ of no difference. We used MS excel for data management and Stata 11.0 for Mac for the analyses. Generally, a two-sided *p*-value < 0.05 was considered statistically significant.

We investigated the effect of manikin characteristics on several measures of CPR quality. We tabulated data according to manikin-group allocation and used the Mann-Whitney-U test for testing the H0 of no difference.

We assessed the influence of age, body size, sex, and prior experience on the effect of manikin characteristics on the measures of CPR-quality using linear regression. For this analysed outcome was each a measure of CPR-quality, co-variables were manikin group allocation as well as the potentially influential variables separately and altogether. We used a robust standard error to allow for the clustered structure of our data. For the outcomes where the distribution was not normal we additionally used bootstrap standard errors for further calculations. For bootstrapping 1000 replications yielded stable estimates.

To investigate effect modification of these potentially influential factors we dichotomized these factors and included these as interaction terms into the above-described models. The Wald-test for the interaction term was interpreted as the test for interaction.

We tabulated age and the frequency of the questionnaire answers to the question “Would you like to repeat the training in the future?” to investigate the effect of age on the willingness to repeat the training in the future and used a Chi^2^ test for hypothesis testing.

## Results

We screened 349 children from two elementary and one middle school and excluded 27 of these (11 were < 8 and 14 were > 13 years old, 2 had a plaster cast). We finally included a total of 322 children. One child turned fourteen at the day between inclusion and testing but was not excluded from participation. There were no dropouts in the study. We randomly assigned 164 children to the soft manikin group and 158 to the standard manikin group. All enrolled children completed the trial. Baseline characteristics and demographics were similar between study groups (Table 1).

**Table 1:** Baseline characteristics and demographics.

Characteristics		Standard manikin group (n = 158)	Soft manikin group (n = 164)
Age, years	< 9	13 (8)	18 (11)
	9 to 10	67 (43)	86 (52)
	> 10 years	78 (49)	60 (37)
Median age, years		10	10
Gender	Male	66 (42)	75 (46)
	Female	92 (58)	89 (54)
Body weight, kg		41.4 ± 11.8	40.1 ± 11.5
Body height, cm		146.9 ± 11.6	144.5 ± 10.9
Native language	German	127 (80)	122 (75)
	Non German	31 (20)	42 (25)
Prior exposure to CPR training	Yes	65 (41)	82 (50)
	No	93 (59)	82 (50)

Data are presented as absolute counts (percentage) or as mean ± standard deviation.

### Primary analysis, motivation

The vast majority of children had fun at the training (standard manikin group 99%, soft manikin group 98%; P = 0.32), were interested in the training (standard manikin group 98%, soft manikin group 99%; P = 0.65), and would like to repeat the training the future (standard manikin group 91%, soft manikin group 89%; P = 0.89).

For all children, it was important to know how to help in case of an emergency.

**Fig 2:**
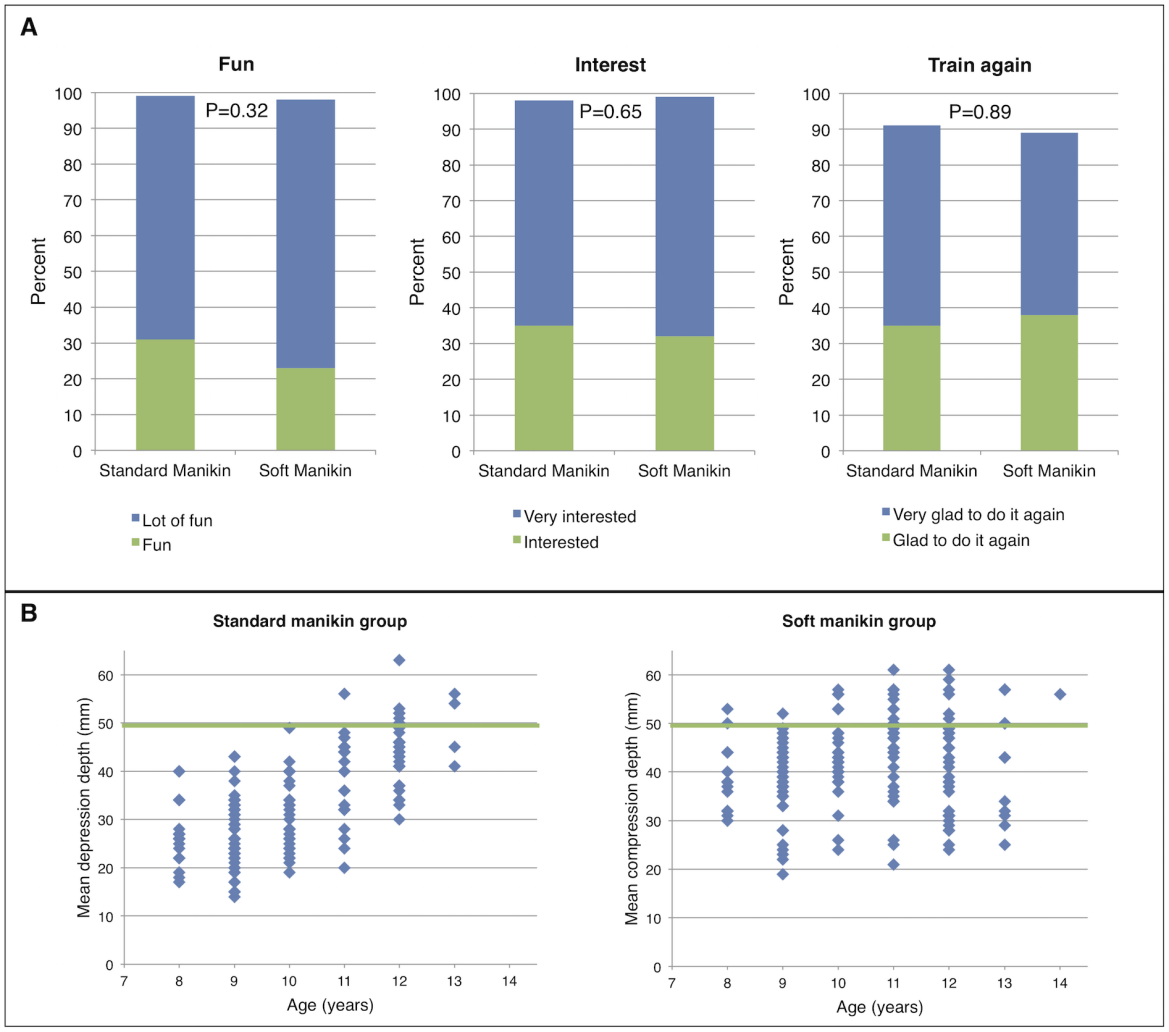
Questionnaire results for children’s fun, interest, and willingness to repeat training in the future (A). Mean chest compression depth by age (B).

Although manikin stiffness affected CPR performance (see section “Primary analysis, CPR performance”), manikin stiffness did seem to affect children´s motivation or interest in the training as there was no significant between-group difference in any of the answers of the questionnaire (Table 2 and Fig 2(A)).

**Table 2:** Questionnaire results.

Question	Possible answers	Standard manikin group (n = 158)	Soft manikin group (n = 164)	P Value
Did you enjoy the training?	Yes, it was a lot of fun	108 (68)	123 (75)	0.32
	Yes, it was fun	48 (31)	38 (23)	
	No, I had little fun	2 (1)	2 (1)	
	No, I didn´t like it at all	0 (0)	1 (1)	
Were you interested in the training?	Yes, I was very interested	99 (63)	110 (67)	0.65
	Yes, I was interested	56 (35)	52 (32)	
	No, I was little interested	3 (2)	2 (1)	
	No, I was not interested at all	0 (0)	0 (0)	
Would you like to repeat the training in the future?	Yes, I would be very glad to do it again	88 (56)	84 (51)	0.89
	Yes, I would like to do it again	55 (35)	62 (38)	
	No, I would rather not do it again	13 (8)	16 (10)	
	No, I definitely don´t want to do it again	2 (1)	2 (1)	
Was it easy for you to perform chest compressions?	Yes, it was very easy	55 (35)	49 (30)	0.39
	Yes, it was easy	69 (44)	84 (51)	
	No, it was difficult for me	33 (20)	28 (17)	
	No, it was very difficult for me	1 (1)	3 (2)	
How good were you performing chest compressions?	I did it very well	61 (39)	73 (44)	0.23
	I did it well	85 (54)	85 (52)	
	I did it not really well	12 (7)	6 (4)	
	I did it very badly	0 (0)	0 (0)	
Do you think it is important to know how to help in cardiac arrest?	Yes, it is very important	150 (95)	154 (94)	0.81
	Yes, it is important	8 (5)	10 (6)	
	No, it is of minor importance	0 (0)	0 (0)	
	No, it is not important at all	0 (0)	0 (0)	

Data are presented as absolute counts (percentage).

### Primary analysis, CPR performance

Although most children felt that performing chest compressions was easy (standard manikin group 79%, soft manikin group 81%; P = 0.39) and also thought they had performed them well (standard manikin group 93%, soft manikin group 96%; P = 0.23), the overall CPR quality, as quantified with the compression score, was generally poor (median compression score 20, IQR 2–49).

However, significant differences between the groups were observed (standard manikin group median compression score 8, IQR 1–45 vs soft manikin group median compression score 29, IQR 11–54; P <0.001).

The mean compression depth, the fraction of compressions fully released, and the fraction of deep enough compressions were better in the soft manikin group compared to the standard manikin group. However, the mean compression rate and the fraction of compressions at an adequate rate was significantly higher in the standard manikin group. No difference in correct hand position between the groups was observed.

A strong positive correlation of the mean compression depth and age was found in the soft manikin group (Pearson correlation coefficient r = 0.69) while only a weak correlation of these parameters was found in the soft manikin group (Pearson correlation coefficient r = 0.16). Further details of the results of CPR performance are presented in Table 3 and Fig 2(B).

**Table 3:** CPR quality characteristics derived from the Laerdal SkillReporter^™^.

Characteristics	Standard manikin group (n = 158)	Soft manikin group (n = 164)	P Value
Compression Score, %	8 (1–45)	29 (11–54)	<0.001
Correct hand position, %	100 (100–100)	100 (99–100)	0.295
Mean compressions depth, mm	33 ± 10	41 ± 9	<0.001
Compression fully released, %	90 (46–99)	97 (84–99)	<0.001
Deep enough compressions, %	0 (0–11)	12 (0–39)	<0.001
Compressions adequate rate, %	23 (6–52)	3 (0–29)	<0.001
Compressions, rate per minute	120 ± 16	137 ± 15	<0.001

Data are presented as median (interquartile range) or mean ± standard deviation.

### Secondary analysis

The effect of manikin´s chest stiffness on how much children enjoyed the CPR training differed between children with prior exposure to CPR training (OR 0.75; CI 0.35 to 1.61), compared with those without prior exposure (OR 2.09; CI 1.08 to 4.06, P for interaction 0.047). This suggests that children without prior exposure to CPR training, allocated to a soft manikin experienced more fun.

**Fig 3:**
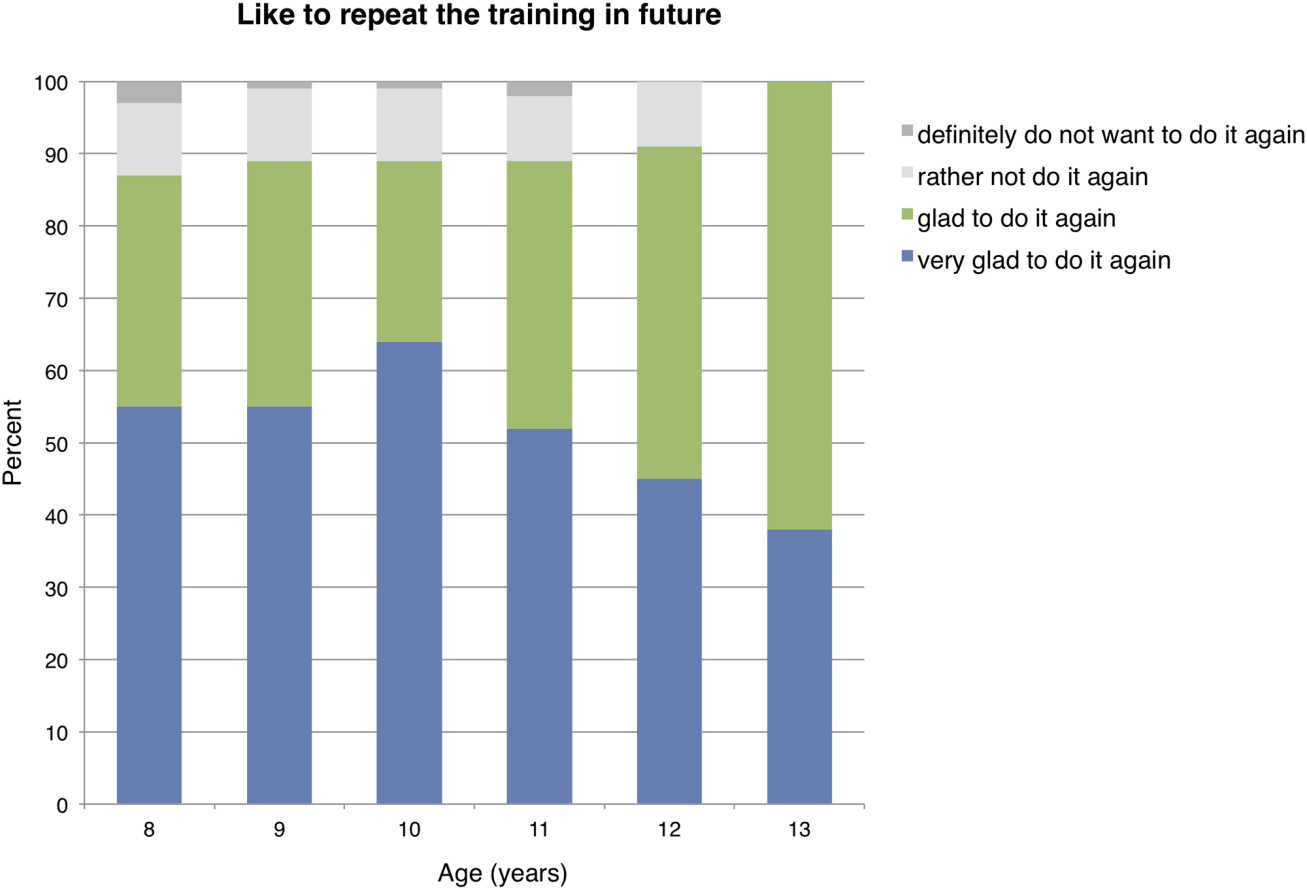
Questionnaire results for children’s willingness to repeat training in the future in different age groups.

The willingness to repeat a CPR training in the future was not significantly affected by age (P = 0.65, Fig 3).

Children with a body weight > 44kg seemed to be more willing to repeat the training when allocated to a soft manikin (OR 1.79; 95%CI 0.84 to 3.84, P for interaction 0.015)

However, since we did not correct these secondary analyses for multiple testing, the significance of results should be interpreted with caution and only considered to be hypothesis generating.

Further details are provided in the supplemental material (S1 Table).

## Discussion

We found that children were not discouraged by CPR training and that the stiffness of the manikin did not affect their motivation after the training. This finding challenges the current thought that younger children should not receive CPR training because they might be discouraged by not being able to adequately perform chest compressions.

There is undisputed evidence that starting CPR training at an early age raises bystander CPR rate and improves survival in OHCA. Accordingly, annual CPR training is recommended for schools world-wide, as reflected in leading specialist societies’ statements [17,27].

However, current guidelines recommend starting CPR training at the age of 12 years only. This is based on the observation that the ability to perform sufficient chest compressions is strongly influenced by children’s weight. A body weight of at least 50 kg was shown necessary to sufficiently compress CPR manikins. This weight is usually reached at the age of 13 years [14,15].

In contrast to the assumptions so far, younger children remained highly motivated and had a positive attitude towards future CPR training in our study. This high motivation was probably more related to the way the training was performed and how trainers gave feedback than to the resistance of the manikin but might further be optimized if children do their first CPR training on soft manikins as in special subgroups of our study this was associated with increased enjoyment.

Without doubt, most of these children would not be able to perform adequate CPR on an adult in a real life scenario because hardly any child was able to adequately compress the manikins’ chest. At the same time, they believed that they did well at performing chest compressions. This discrepancy in self-assessment and actual performance is common in children and improves with increasing age and experience with school [28]. However, the point of training children in CPR is not to make them a perfect “resuscitationist” instantly but to set the basis for future trainings and to allow them spreading their knowledge and motivation about CPR to their family members and to the society.

Ideally, first aid providers are characterized by the following four conjoined attributes: *knowledge* about BLS, *awareness* about the importance of BLS, *motivation* to help and start BLS, and the *physical ability* to perform high quality BLS.

Children can achieve a profound *knowledge* about BLS and retain good knowledge even after months [19,29]. Interestingly, in our study children’s *motivation* and *awareness* after training were higher compared to findings in average adults [30].

It is only the lack of *physical ability* in younger children to perform effective chest compressions to fulfil all criteria of an ideal first aid provider. These children can gain the knowledge, are highly motivated and aware of the importance of CPR and as a matter of fact the physical ability will develop by time as they grow up. Altruism research has shown that pre-pubertal children have a less inhibited approach to resuscitation training [12,17]. This might also be supported by our finding that children with a body weight > 43 kg reported less frequently to be “very glad to do the training again”, assuming these children were already closer to puberty. Consequently, considerable effort should be made to build knowledge, awareness, and motivation already at elementary schools to promote BLS when physical ability emerges. One might argue, that a social desirability bias may eventually explain the positive attitudes towards CPR training in these children, especially as many of them reported a prior exposure to CPR training. However, this information comes from written questionnaires completed by the children individually, anonymously and in a protected place. Therefore, we believe, that at the best the positive atmosphere during these exercises at the schools could explain this effect, which is however already part of the intended intervention. The Austrian government recommends that every child should receive first aid training during their school career and as a consequence such trainings are offered at all stages of school but CPR is only superficially covered in these trainings. Given the results of our study, we are convinced that hands on CPR training should also be included in these trainings. Concerns of discouraging children by exposing them to CPR training are not justified.

Another limitation might be a training duration of 30 min. in our study, which is not in line with the WHO recommendations of 2 hours of training for 12-year-old children. However, there is no scientific data regarding training motivation or the ability of 12 year old or younger children to perform chest compressions and the recommended 2 hours of training contain other skills besides CPR training and the main concern was that children might be discouraged especially by CPR, which has been refuted even during our 30 minute training. The reason why the guidelines do not recommend training for children younger than 12 is that it has been anticipated that children might not be able to be trained in CPR which has however been clearly challenged by our data. As always in such experiments we cannot exclude a Hawthorne effect, which may explain enhanced performance under observation or test situations. However, improved survival rates in countries where regular resuscitation training is provided speak in favour of a strong effect on real-life utilization of acquired skills. Overall, we believe that the children’s high effort during training might be an indicator of true motivation.

In conclusion, our results refute the view that children are discouraged or uninterested when receiving CPR training just because they are not able to perform adequate chest compressions on a stiff manikin. Training on a manikin with a reduced resistance did not have any effect on motivation after a CPR training in this study. However, despite the high motivation, the actual CPR performance of young children was generally poor in our study. The fact that young children are physically not able to adequately perform CPR might be a limiting but self-resolving issue as children get stronger while they grow. We therefore think that authorities should recommend and implement CPR training in schools for children aged 8 years or older to set the basis for their career as future “resuscitationists”.

## Supporting information

S1 Questionnaire (DOCX)

S1 Table Interaction of soft manikin group allocation and prior CPR exposure/body weight on motivation. (DOCX)
