# Supplementary material for: The impact of cardiopulmonary resuscitation (CPR) manikin chest stiffness on motivation and CPR performance measures in children undergoing CPR training—A prospective, randomized, single-blind, controlled trial
Source: PLoS One. 2018 Aug 16;13(8):e0202430. doi: 10.1371/journal.pone.0202430 (PMC6095555; doi:10.1371/journal.pone.0202430)
Supplement: S1 Questionnaire — (DOCX) [file pone.0202430.s001.docx]

*Questionnaire*

1. Did you enjoy the training?

1. Yes, it was a lot of fun.
2. Yes, it was fun.
3. No, I had little fun.
4. No, I didn´t like it at all.

2. Were you interested in the training?

1. Yes, I was very interested.
2. Yes, I was interested.
3. No, I was little interested.
4. No, I was not interested at all.

3. Would you like to repeat the training in the future?

1. Yes, I would be very glad to do it again.
2. Yes, I would like do to it again.
3. No, I would rather not do it again.
4. No, I definitely don´t want to do it again.

4. Was it easy for you to perform chest compressions?

1. Yes, it was very easy.
2. Yes, it was easy.
3. No, it was difficult for me.
4. No, it was very difficult for me.

5. How good were you at performing chest compressions?

1. I did it very well.
2. I did it well.
3. I did not do it really well.
4. I did it very badly.

6. Do you think it is important to know how to help people in cardiac arrest?

1. Yes, it is very important.
2. Yes, it is important.
3. No, it is of minor importance.
4. No, it is not important at all.
