# Supplementary material for: The impact of cardiopulmonary resuscitation (CPR) manikin chest stiffness on motivation and CPR performance measures in children undergoing CPR training—A prospective, randomized, single-blind, controlled trial
Source: PLoS One. 2018 Aug 16;13(8):e0202430. doi: 10.1371/journal.pone.0202430 (PMC6095555; doi:10.1371/journal.pone.0202430)
Supplement: S1 Table — (DOCX) [file pone.0202430.s002.docx]

S1_Table Interaction of soft manikin group allocation and prior CPR exposure/body weight on motivation

| Question | Characteristics | Answer | Standard manikin group (n=158) | Soft manikin group (n=164) | OR (95% CI)* | P |
| --- | --- | --- | --- | --- | --- | --- |
| Did you enjoy the training? | All children | "yes, it was a lot of fun" | 108 (68) | 123 (75) | 1.39 (0.88 to 2.18) | 0.047 |
|  |  | "it was fun" to "I didn’t like it at all" | 50 (32) | 41 (25) |  |  |
|  | Children with prior exposure to CPR training | "yes, it was a lot of fun " | 51 (79) | 60 (73) | 0.75 (0.35 to 1.61) |  |
|  |  | "it was fun" to "I didn’t like it at all" | 14 (22) | 22 (27) |  |  |
|  | No prior exposure to CPR training | "yes, it was a lot of fun " | 57 (61) | 110 (67) | 1.21 (0.78 to 1.88) |  |
|  |  | "it was fun" to "I didn’t like it at all" | 36 (39) | 54 (33) |  |  |
|  |  | “I was interested” to “not interested at all” | 59 (37) | 54 (33) |  |  |
| Would you like to repeat the training in the future? | All children | “I would be very glad to do it again” | 88 (56) | 84 (51) | 0.83 (0.50 to 1.40) | 0.015 |
|  |  | “I would like to do it again” to „Definitely don´t“ | 70 (44) | 80 (49) |  |  |
|  | Participants weight 21 to 33 kg | “I would be very glad to do it again” | 32 (64) | 29 (51) | 0.58 (0.27 to 1.26) |  |
|  |  | “I would like to do it again” to „Definitely don´t“ | 18 (36) | 28 (49) |  |  |
|  | Participants weight 34 to 43 kg | “I would be very glad to do it again” | 35 (67) | 27 (51) | 0.50 (0.23 to 1.11) |  |
|  |  | “I would like to do it again” to „Definitely don´t“ | 17 (33) | 26 (49) |  |  |
|  | Participants weight 44 to 87 kg | “I would be very glad to do it again” | 21 (38) | 28 (52) | 1.79 (0.84 to 3.84) |  |
|  |  | “I would like to do it again” to „Definitely don´t“ | 35 (62) | 26 (48) |  |  |

Data are presented as absolute counts (percentage). * 95% CI denotes 95% confidence interval and is calculated on the basis of robust standard errors to allow for the cluster structure of data. P indicates the P value for interaction.
